# Visualizing Vertebrate Embryos with Episcopic 3D Imaging Techniques

**DOI:** 10.1100/tsw.2009.154

**Published:** 2009-12-16

**Authors:** Stefan H. Geyer, Timothy J. Mohun, Wolfgang J. Weninger

**Affiliations:** ^1^ Centre for Anatomy and Cell Biology, Medical University of Vienna, London, UK; ^2^ Developmental Biology Division, MRC National Institute for Medical Research, London, UK

**Keywords:** 3D modeling, imaging, embryo, episcopic microscopy, development

## Abstract

The creation of highly detailed, three-dimensional (3D) computer models is essential in order to understand the evolution and development of vertebrate embryos, and the pathogenesis of hereditary diseases. A still-increasing number of methods allow for generating digital volume data sets as the basis of virtual 3D computer models. This work aims to provide a brief overview about modern volume data–generation techniques, focusing on episcopic 3D imaging methods. The technical principles, advantages, and problems of episcopic 3D imaging are described. The strengths and weaknesses in its ability to visualize embryo anatomy and labeled gene product patterns, specifically, are discussed.

## INTRODUCTION

Vertebrates are a subphylum (subdivision) of chordates and include mammals, birds (Aves), reptiles, amphibia, and fish (Chondrichthyes, Agnatha, Osteichthyes). As in all eukaryote diploid organisms, their early ontogenetic stages are termed embryos. The embryonic period starts with the first cleavage of the zygote, and ends with hatching or metamorphosis in birds, reptiles, amphibia, and fish.

In mammals, the embryonic period is only the first period of intrauterine life, in which the cells of the developing organism undergo differentiation and specialization and form organs. The second period of intrauterine life is named the fetal period and lasts until birth (hatching in Protheria). The relative length of the mammalian embryonic period varies between species. In the human (10 lunar months of intrauterine development), it lasts 8 weeks (this approximately corresponds to the first 10 weeks of pregnancy). In the mouse (21 days of intrauterine development), it lasts approximately until E14 ($14^{th}$ day of pregnancy). However, we like to emphasize that most modern developmental biologists simply use the term “embryo” when referring to unborn animals.

## The Need for Virtual Embryos

Precise three-dimensional (3D) visualization of the morphology of vertebrate embryos and of gene products in the context of their tissues is important in order to understand the mechanisms that drive embryogenesis. For three reasons, such knowledge is of the utmost importance:

1. Precise knowledge about the mechanisms driving ontogenesis in different species is essential for researching the evolution of vertebrate development[1,2,3,4,5,6,7].
2. Precise 3D and 3D-based four-dimensional (4D) visualizations of normal embryo development are valuable tools for teaching embryology to students, graduates, and postgraduates[8].
3. Embryos of several vertebrate species (*Mus musculus*, *Gallus domesticus*, *Coturnix coturnix*, *Xenopus laevis*, and *Danio rerio*) are popular model organisms for researching the genesis of human hereditary diseases. Gene product patterns in the context of tissues and the morphological phenotype of genetically engineered or experimentally manipulated embryos are analyzed and compared to normally developed littermates of the same embryonic stages[9,10,11,12,13]. This approach leads to a better understanding of the genetic and epigenetic regulation of morphogenetic events, and eventually will pave the way to new diagnostic strategies and therapeutic tools.

## The Need for High-Resolution Volume Data

Researching the precise cellular arrangement, the tissue architecture, and the topology of patterns of gene products in the context of organs and organ systems of developing embryos requires the availability of digital volume data of high spatial resolution. The same is true for morphometric analysis of blood vessels and other embryonic structures[14].

The resolution of a digital volume data set is usually defined by the size of its voxels. Voxels are the cubes, or cuboids, that comprise a volume data set as its smallest discrete entities. The smaller its voxels, the better the “numerical” resolution of a volume data set. However, this might prove misleading, since the numerical digital volume data resolution, as defined by the voxel size, is not the same as the true or spatial resolution of this volume data. In contrast to the numerical resolution, spatial resolution is defined as the shortest distance between two points that can be distinguished as separate entities. Only the spatial resolution defines to which size small anatomical details can be observed and how significant morphometric analyses are.

Many 3D data-generation techniques are capable of creating data of high numerical resolution (with small voxel sizes). However, these data might be of poor spatial resolution. For example, histological section-based and episcopic 3D data-generation methods use digital cameras sitting on microscopes for volume data generation. Modern cameras and small section thicknesses easily permit the creation of volume data with voxel sizes as small as $1 \times 1 \times 1$ µm. However, if such data are created using an objective, the numerical aperture of which limits the spatial resolution to 2 µm, the effective resolution of the data is lower than $2 \times 2 \times 1$ µm. The higher numerical resolution is worthless.

Besides the optical components, other factors influence the spatial resolution. During the generation of digital 3D data, interpolation errors[15] or partial-volume artefacts[16,17] are introduced, meaning that the content of one voxel is not strictly assigned to a certain anatomical structure. As a consequence, the shortest distance between two points that can be distinguished as separate entities is larger than one voxel. Hence, in most volume data sets, the spatial resolution is lower than the numerical resolution.

Applying this definition of spatial resolution, even the accuracy of the depiction of cell and tissue borders influences the resolution of a volume data. Vague definitions of cell or tissue borders, as it often is the case in data obtained by utilizing tissue (auto-) fluorescence for visualization, negatively influence spatial resolution. The same is true for inappropriate signal to noise ratios in MRI images. Again in such volume data, the spatial resolution is lower than the numerical resolution.

## Volume Data–Generation Methods – a Brief Overview

The microscopic anatomy of vertebrate embryos cannot be adequately explored simply by anatomical dissection. Therefore, it was the use of histological sections that first allowed more detailed insights into embryo morphology. These can show extremely high detail at the cell, tissue, and gross anatomical levels.

The quality of digital images captured from histological sections is also very high. No modern 3D imaging technique, except for the high-resolution episcopic microscopy (HREM) method (see below), is capable of producing digital data that approach the quality of a digital image captured from a histological section.

## Histological Section-Based 3D Imaging Methods

Embryos are very complex 3D objects and histological sections only provide two-dimensional (2D) information. The logical consequence for early embryologists living at the end of the 19th century was to develop techniques for reconstructing physical 3D representations of the inner structures of embryos out of a series of sequential histological sections[18,19,20]. Since that time, 3D reconstruction techniques based on histological section series have been steadily modernized and optimized. Even today, sophisticated histological section-based 3D imaging methods are still in use[21,22,23,24,25].

However, 3D reconstruction techniques utilizing histological sections have severe disadvantages. First, histological sections show various nonaffine artefacts. These are introduced during cutting of the sections, their stretching on the surface of water, and their subsequent mounting on glass slides. These artefacts alter unpredictably the topology and the extension of anatomical structures. Second, such digital volume data sets are created by capturing digital images manually, in a “section by section” approach. (Section digitalization must be performed with a digital camera sitting on a compound microscope, since high throughput section scanners cannot capture images of high spatial resolution.) Third, the single digital images of a digitized histological section series are not aligned properly. They have to become realigned, which is a major technical challenge. Different approaches have been developed for semiautomated and automated section realignment[22,23,26,27,28,29,30,31,32,33,34,35,36,37]. Although they can lead to reasonable results, they are time expensive and error prone. For these reasons, histological section-based 3D reconstruction techniques are of limited and specialist use[24,38,39].

## 3D Imaging Methods that Instantly Produce Digital Data

In the last decades, many techniques have been developed, which, in addition to other tasks, permit the immediate creation of digital volume data of vertebrate embryos, their organs, tissues, and cells. They include ultrasound (US, e.g., [40,41]), microcomputed tomography (μCT, e.g., [42,43]), micromagnetic resonance imaging (μMRI, e.g., [44,45]), optical coherence tomography (OCT, e.g., [46,47]), optical projection tomography (OPT, e.g., [48,49]), confocal microscopy (e.g., [50,51]), thin-sheet laser imaging (e.g., [52]), digital scanned laser light sheet fluorescence microscopy (e.g., [53]), selective plane illumination microscopy (e.g., [54]), two-photon fluorescence microscopy (e.g., [53]), episcopic 3D imaging (see below), histological section-based 3D reconstruction techniques (e.g., [55]), electrontomography (e.g., [56,57]), and serial block-face scanning electron microscopy (e.g., [58]).

Some techniques, such as US[40,59,60], μMRI[61,62,63,64,65,66,67], OCT[68,69,70,71], OPT[72], and confocal microscopy[73] permit *in vivo* 3D visualization of embryo anatomy and/or 4D analysis of ontogenetic events. Some methods, such as μMRI, OPT, and confocal microscopy even attempt *in vivo* 3D analysis of gene expression data[62,72,74].

However, in *vivo* imaging of embryos is still experimental. Due to motion artefacts and technical problems, their resolution is rather poor and most imaging modalities are chiefly used as postmortem methods for analyzing sacrificed embryos (Table 1). Postmortem μCT[75,76], μMRI[77,78,79,80,81, 82,83], and OPT[84,85,86,87,88,89,90] are capable of producing volume data of embryos in short scanning times and high quality. Still, their spatial resolution is too low to permit 3D reconstruction of cells and small anatomical details. On the other hand, methods such as postmortem optical sectioning techniques and electron microscopic techniques produce data that are of high cellular and subcellular detail[91,92,93,94]. However, this detail cannot be visualized in the 3D context of larger embryos and their organs.

**TABLE 1**
**3D Imaging Techniques for Visualizing Vertebrate Embryos***

| | Resolution | Detection of Specifically Stained Structures | Advantages | Drawbacks |
|---|---|---|---|---|
| US | $30 \times 30 \times 30$ µm$^3$ | No | Comparably cheap | Low contrast |
| µMRI | $25 \times 25 \times 25$ µm$^3$ ($10 \times 10 \times 10$ µm$^3$) | Not routinely | High throughput scans possible | Partial volume artefacts, limited field of view |
| µCT | 8 x$\times$ 8 $\times$ 8 µm$^3$ ($1 \times 1 \times 1$ µm$^3$) | No | Table scanners commercially available | Limited field of view |
| OPT | $5 \times 5 \times 5$ µm$^3$ | Yes | Multiple stainings possible | Only small and transparent embryos, whole mount staining required |
| Histological section series | $0.3 \times 0.3 \times 7$ µm$^3$ | Yes | Multiple stainings possible, high-resolution 2D section images | Labor intensive, time expensive, limited z-resolution (section thickness) |
| Confocal microscopy | $0.3 \times 0.3 \times 0.3$ µm$^3$ | Yes | Fast, multiple stainings possible | Only small specimens, low contrast of unstained tissues |
| Episcopic microscopy | $0.5 \times 0.5 \times 1$ µm$^3$ | Yes | Cheap, high contrast of unstained tissues | Whole mount staining required, shining through artefacts |

*Note:* The table does not provide the minimal resolution, which is technically feasible, but the resolution that can be obtained with biological material.

The gap between high resolution "out of organ context imaging" and "low resolution imaging" of entire embryos is closed by a heterogeneous group of techniques we have subsumed under the term "episcopic 3D imaging methods". All of them are suitable for visualizing tissues and small anatomical details in the context of embryonic organ systems.

## EPISCOPIC 3D IMAGING METHODS

Episcopic imaging is defined as the capturing of an image of the surface of a 3D object. This definition includes everything that creates 2D images by depicting light waves or electrons that are reflected or emitted from physical objects, thereby including traditional photography and surface electron microscopy[95]. However, neither traditional photography nor SEM can be used for creating digital volume data. Therefore, we introduce the term "episcopic 3D imaging" and use it for summarizing methods, which, at the light microscopic level, episcopically generate volume data from histologically embedded specimens during their sectioning. The following sections will outline the principle workflow, describe the technical challenges of epsicopic 3D imaging in general, and discuss the specifics of the various existing methods.

## Equipment and Workflow

Episcopic 3D imaging techniques are "destructive postmortem" imaging techniques. They create volume data by physically destroying harvested and fixed embryos. The principle workflow of generating episcopic volume data is rather simple:

After harvesting, the embryos are processed and embedded in histological embedding material. The histological blocks are mounted on the episcopic data-generation apparatus. An episcopic data-generation apparatus essentially combines a microtome, a PC-driven digital video camera sitting on the phototube of a fluorescence microscope, and gadgets for aligning the optics with the microtome (adjustable x,y stages, etc.). If a rotary microtome is part of the data-generation apparatus, the optical path of the microscope is aligned with a position at which the block holder arm of the microtome comes to rest after each cut. If a sliding microtome is part of the data-generation apparatus, the optical path of the microscope is aligned either with the stepwise ascending block or with a photo position to which the block is shifted after each cut. All currently existing data-generation apparatuses are still prototypes.

The process of data generation is as follows: single digital images of the surface of the block are captured using each excitation/emission wavelength combination of interest. Then the block surface is removed by cutting or milling, and the process is repeated with the freshly exposed block surface. This process is reiterated until the block is entirely sectioned and a series of digital images representing the entire block is created. Since all images of a series are inherently aligned, they can be simply stacked together and converted into a volume data set. The x-,y-dimensions of the pixels provide the x- and y-dimensions of the voxel. The distance between two subsequent images provides the z-dimension of the voxel.

In principle, all episcopic 3D data-generation techniques permit capturing of multiple overlapping images of the block surface. These images can be merged ("stitched" together), thereby expanding the field of view, while at the same time retaining high resolution. However, this process dramatically increases data-generation time.

Once the data are created, they are processed, visualized, and analyzed with the aid of commercially and freely available software packages. Software tools allow for the creation of straight, oblique, and curved virtual resections through the volume data, maximal and minimal intensity projections, and the creation of volume-rendered 3D models. As an alternative to volume rendering, they also permit filtering, contrast enhancement, and automatic, semiautomatic, and manual thresholding of interesting embryonic structures as preparation for surface rendering. In addition to 3D visualization and topological analysis of computer models, they usually permit quantitative analysis.

## Technical Aspects of Episcopic 3D Imaging

This section aims to briefly outline the main specific requirements of episcopic 3D imaging techniques in general.

### *Orientation of the Embryo in the Block*

An episcopically captured data volume has the form of a cuboid. One of its sides is the area of the block surface that the digital camera captures (field of view). Episcopic 3D imaging only digitizes those parts of an embryo that are located within the data cuboid beneath the field of view.

High magnifying objectives, as necessary for capturing high-resolution data, come along with small fields of view. In order to be able to define a small field of view, it is essential to embed the embryo in a proper orientation into the data cuboid. This requires that the embedding medium remains transparent during the embedding process. Consequently, episcopic 3D imaging methods, which employ transparent embedding media, can generate volume data of a higher spatial resolution than methods that employ opaque embedding media.

### Definition of the Field of View

Before data capturing is started, the field of view must be carefully defined to permit its precise alignment with the optical path of the microscope. The easiest way to define the size and position of the field of view is to project the silhouette of the embryo resting inside the block towards its surface. Again, this can be best achieved if transparent embedding media are used.

### Elimination of Shining Through Artefacts

Besides achieving sufficient tissue contrast, the greatest challenge for episcopic 3D imaging is to capture information from the block surface only. The “shining through” of information, stemming from tissues located inside the block to its very surface, must be reduced.

Several approaches have been developed for reducing shining through artefacts. The simplest approach is the inclusion of dyes in the embedding medium. Episcopic 3D imaging techniques utilizing tissue fluorescence or whole mount tissue contrasting employ this approach (Fig. 1). In contrast, on block staining techniques profit from the poor contrasts of the yet-unstained tissues of the embedded specimen, using digital subtraction of the unstained block surface from the corresponding image of the stained block surface (see below).

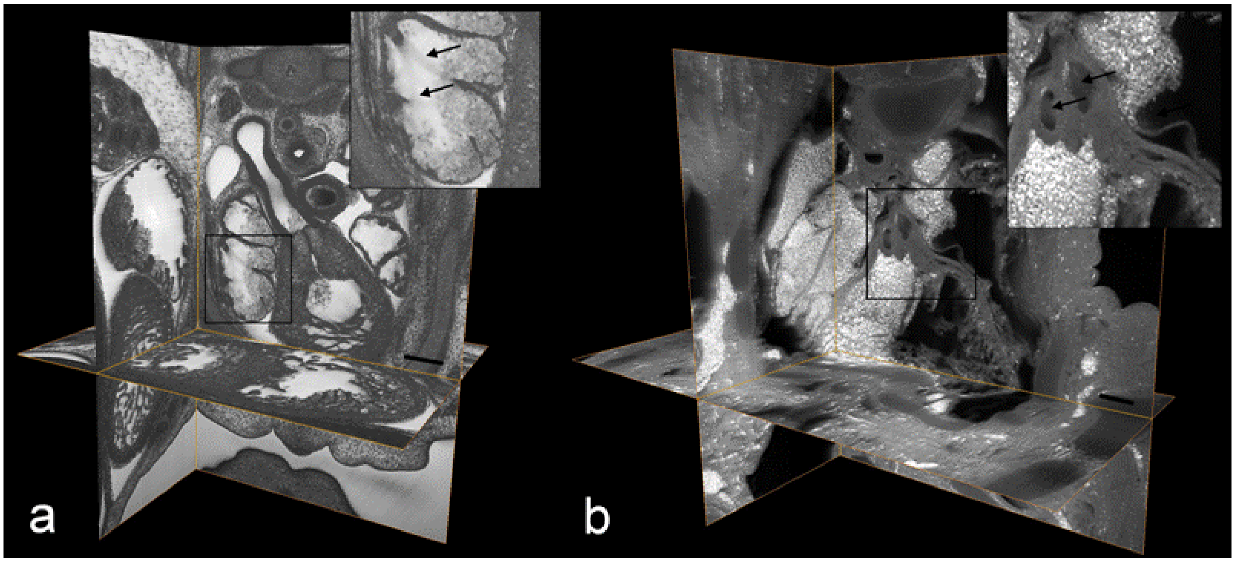

**FIGURE 1.** Shining through artefacts in HREM volume data (a) and EFIC volume data (b) of thoraces of 14.5 dpc mouse embryos. Both data were generated from quickly produced blocks and show regionally enhanced shining through artefacts especially in the cardiac chambers and the great intrathoracal arteries (arrows in inlays). Careful block preparation reduces the extension of the artefacts (Fig. 2). Scale bars, 200 µm.

## Episcopic 3D Data-Generation Methods

Various episcopic 3D imaging methods have emerged in the last few years. All of them suggest different approaches for solving the problems inherent to episcopic imaging. Two of these techniques have been named as “surface imaging microscopy” (SIM). In order to avoid confusion, we will refer to the method that was published earlier as $SIM_1$ and to the method published later as $SIM_2$.

Depending on the method used for achieving tissue visualization, episcopic 3D imaging techniques can be grouped into four groups (tissue bleaching methods, on block staining methods, tissue fluorescence methods, and whole mount eosin staining methods). This section aims to describe the characteristics of each method briefly, and discuss their strength and limits. Table 2 lists research publications that have used episcopic 3D imaging techniques.

**TABLE 2**
**Nonmethodological Scientific Publications in which Episcopic 3D Imaging Methods were Applied**

| | Visualization of Tissue Architecture | Embedding Medium | Minimal Section Thickness | Detection of Specifically Labeled Gene Products | Applications |
|---|---|---|---|---|---|
| Epi-3D | On block staining (lead acetate/sodium sulfide) | Wax, Vybar mixture (semitransparent) | 6 µm | No | [106,107,111] |
| $SIM_2$ | On block staining (adapted histological stains) | Wax (semitransparent) or resin (transparent) | 0.2 µm (resin); 2.5 µm (wax) | Possible; no example yet | |
| EFIC | Autofluorescence | Reddish dyed wax mixture (opaque; transparent during embedding) | 1 µm | Yes (by extinction of autofluorescence) | [108,109,110] |
| $SIM_1$ | Fluorescence | Black polymere (opaque) | 1 µm | Possible; no example yet | |
| HREM | Eosin staining | Reddish dyed resin (transparent) | 0.5 µm | Yes, bluish stained cells and tissues | [14] |

## *Tissue Bleaching Methods*

In 1990, Odgaard et al. published an episcopic 3D imaging method[96] that permitted 3D visualization of the density of the spongiosa trabecles of vertebrae[97,98,99]. The bones are bleached and embedded in black resin. This results in volume data of high contrast and eliminates shining through artefacts. Although this simple approach is highly effective, it is not suitable for visualizing embryos. Hence, it will not be discussed in detail in this paper.

## *On Block Staining Methods*

The characteristic of on block staining methods is the use of histochemical dyes for achieving tissue visualization directly on the block surface. These methods do not permit selective visualization of specifically labeled gene products in their surrounding tissues. The utilized embedding media are transparent, wherefore the embryos can be oriented properly and the field of view can be defined exactly. The methods permit 3D visualizations of the tissue architecture and anatomy of early to late vertebrate embryos of all species.

Three main problems hinder routine applications of on block staining techniques. First, on block staining requires the administration of staining solutions to the surface of the mounted block after each section. This complicates and slows down data generation and hampers its automation. Second, captured block-face images essentially show the reflections of white (polychrome) light, illuminating the tissues on the block surface. Hence, they also show reflection artefacts, which are caused by the scattering of light due to the unevenness of the block surface. Cover slips eliminate these artefacts, but cover slipping dramatically increases the personnel and time expense for data capturing. Third, variations in staining times and dye concentrations effectively lead to a series of slightly inhomogeneous digital images. This inhomogeneity hampers digital data processing and 3D visualization with the aid of volume-rendering algorithms.

Currently two on block staining techniques do exist – Epi-3D and $SIM_2$.

- **Epi-3D**[100] is based on a staining technique developed as early as 1947[101]. Fixed embryos are dehydrated in ethanol containing lead acetate, which accumulates in the embryo and "prestains" its tissues according to tissue density. No color reaction is provoked by the prestaining procedure. The embryos can be embedded in wax or resin blocks, although the best results are achievable by using an embedding mixture composed of stearin, paraffin, and a high percentage of Vybar. The blocks are mounted on the episcopic data-generation apparatus and sectioned. Two digital images of the block surface are captured and on block staining is performed. First, an image that shows the block face as it appears immediately after cutting is captured. Then, sodium sulfide solution is administered to the block surface and, within a fraction of a second, provokes a brownish color reaction due to the formation of lead sulfide precipitate in the tissue. A second image is then captured showing the stained tissues on the block surface and the microtome is triggered to remove the surface section. This process is repeated until the block is entirely sectioned. After completion, the images of the unstained block surfaces are subtracted from the corresponding images showing the stained block surfaces and the resulting image series is converted to a volume data set.
- **$SIM_2$**[102] is a relatively novel invention. Embryos are embedded in wax or resin blocks. The tissues on the block surface are exposed, prepared for staining, and stained with traditional histochemical stains for around 20 sec. After several washing and differentiation steps, a digital image of the block surface is captured. The entire time required for this treatment of the block surface varies with the size of the embryo, but takes approximately 90 sec. After on block staining and image capturing, the uppermost part of the block is removed with the aid of an ultramiller and the staining, image capturing, and milling procedures are repeated until the embedded specimen is entirely sectioned. The resulting image series is converted to a volume data set.

## Tissue Fluorescence Methods

Two episcopic 3D imaging methods have been developed. Both were published in 2002 and utilize the fluorescence of embryonic tissues for achieving unspecific contrasts; episcopic fluorescence image capturing (EFIC)[103] and $SIM_1$[104]. In principle, both EFIC and $SIM_1$ offer the possibility of visualizing gene products in their tissue context; however, due to technical limitations, this is not yet a routine application.

Embryos of all developmental stages and species can be visualized. However, $SIM_1$ has its strength with small embryos, since they can be penetrated rather easily with fluorescence substances. On the contrary, EFIC has its strength with older embryos, since autofluorescence increases with the degree of cell differentiation. EFIC imaging of early embryos can be problematic due to the low amount of tissue autofluorescence in early developmental stages. With both $SIM_1$ and EFIC, specimen orientation and the definition of the correct field of view are problematic, while data generation itself is rather simple and can be fully automated. Compared to most alternative 3D imaging techniques, the quality of the EFIC and $SIM_1$ volume data is high. However, the use of fluorescence signals for detecting tissues may result in relatively diffuse tissue borders. This can lower the spatial resolution of the volume data (see above).

- **EFIC**[103] utilizes the autofluorescence of cells and tissues for achieving tissue visualization and the extinction of autofluorescence, as resulting from *in situ* staining methods for visualizing gene expression patterns. Embryos are prepared as for wax embedding, but infiltrated with and embedded in a special embedding mixture comprising paraffin wax containing Vybar, stearin, and Sudan IV dye. The dyed embedding medium minimizes shining through effects, by suppressing fluorescence emanating from tissues beneath the block surface. During infiltration and embedding, the EFIC wax mixture is hot and remains transparent. This permits proper orientation of the embryo. The cooled and hardened blocks are opaque, thereby hindering the definition of a suitable field of view. To overcome this problem, a grid is placed at the bottom of the mold during the embedding process and a picture of the oriented embryo is captured while the wax is still hot and transparent. After hardening of the block, this picture is used for defining the outlines of the silhouette of the embryo and the correct field of view on the block surface.

- **$SIM_1$**[104] employs fluorescent substances that bind to nucleic acids for achieving unspecific tissue contrasts. In principle, $SIM_1$ offers 3D visualization of specifically labeled gene products, although there are no examples yet. Processing embryos for $SIM_1$ starts with soaking the embryos with the fluorescent substances. Then the embryos are dehydrated and embedded in a black embedding medium, which highly effectively blocks shining through artefacts. However, due to its opacity, the embryos cannot be oriented. Projecting the silhouette of the embryo onto the block surface for defining the field of view is virtually impossible. Standardized embedding molds of various sizes are used in order to overcome this problem.

## *Eosin Whole Mount Staining Methods*

This group only comprises one method. The HREM technique[105] utilizes whole mount eosin staining for achieving tissue visualization. Embryos of all developmental stages and of all species can be visualized. 3D visualization of specifically labeled gene products with bluish color reactions is possible, as long as the embryos are small enough to allow whole mount staining. The HREM data-generation process is fully automated. It creates a large series of digital images near the quality of images of digitized histological sections (Fig. 2) within a few hours. Using high-end digital cameras, spatial volume data resolution is only limited by the section thickness and the numerical aperture of the optical components of the HREM apparatus.

For HREM data generation, embryos are fixed and dehydrated. They are then infiltrated with and embedded in resin dyed with eosin (optionally with related dyes such as acridine orange). The transparency of the reddish dyed resin facilitates proper orientation of the embryo, and exact definition of the size and position of the field of view. Dyeing of the resin has two effects. First, it stains the embryonic tissues in a nonspecific manner. Second, it modifies the inherent fluorescence of the resin around the GFP excitation and emission wavelengths. Therefore, block-face images captured with a GFP or YFP filter set show the eosin-contrasted tissues located at the block surface, while the eosin-dyed resin minimizes shining through of tissue information stemming from beneath the block surface (Fig. 3).

Capturing volume data allowing for 3D visualization of specifically labeled gene products requires the combined use of a YFP and a TX-2 filter set[105]. From each block surface, two corresponding images, one with the YFP and one with the TX-2 filter set, are captured (http://www.meduniwien.ac.at/3D-Rekonstr/HREM/). This results in two series of corresponding images, one comprised of images capturing tissue architecture, the other comprised of images in which the specifically stained tissues are enhanced. The series are processed separately and combined as volume data sets, thereby revealing the precise location of gene products within the embryonic tissues and organs (Fig. 4).

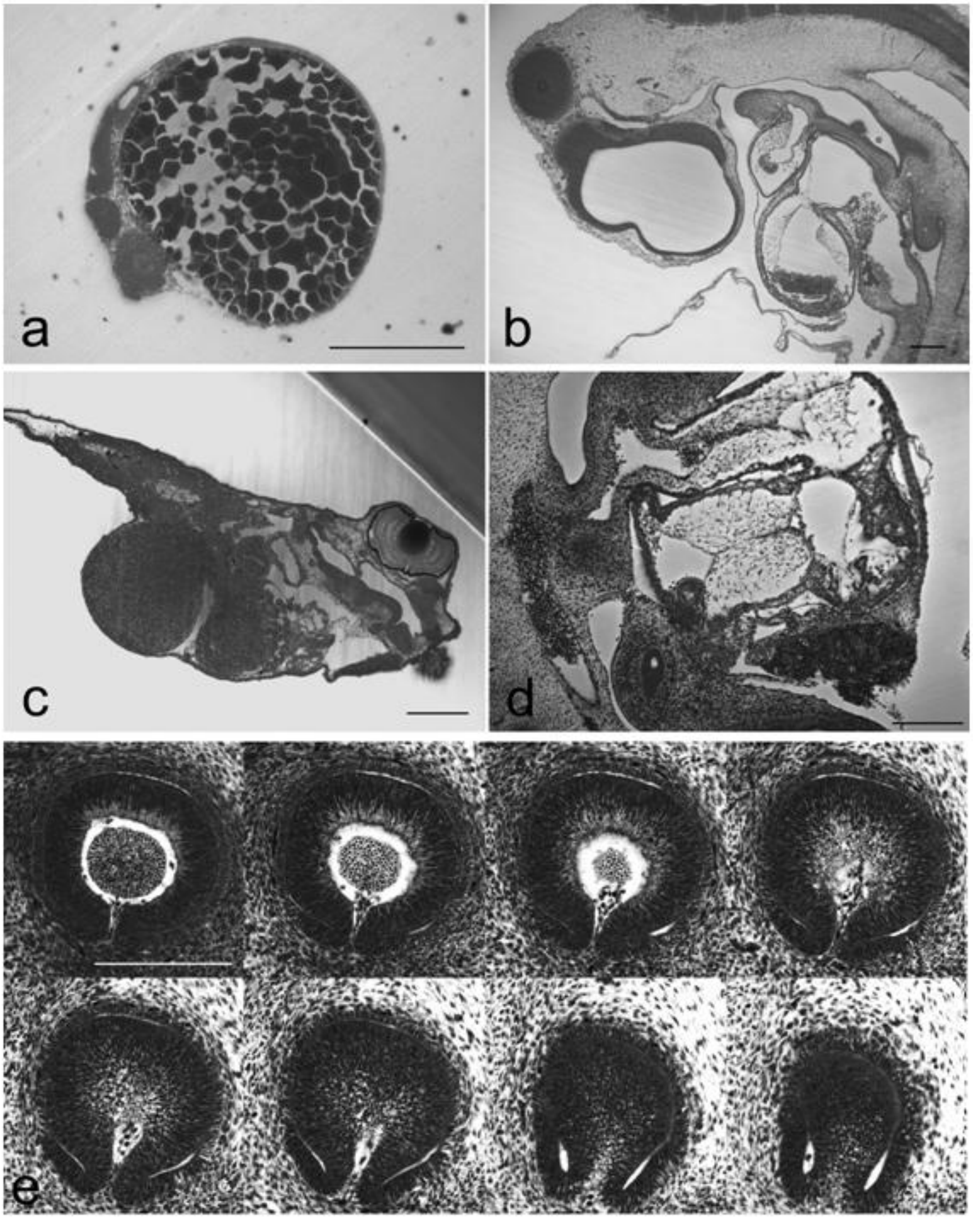

**FIGURE 2.** Digital images captured with HREM. (a) Zebrafish embryo (24–28 h); sagittal section. Note the voluminous yolk sac. (b) Heart and forebrain of a quail embryo (HH20); sagittal section. (c) *Xenopus* embryo (developmental stage 41); oblique section. (d) Heart of a mouse embryo (TS 16); sagittal section. Note the endocardial cushions. (e) Series of HREM images of sections through the eye of a mouse embryo (TS 16). Note that not all sections of the series are displayed. Scale bar, 200 μm.

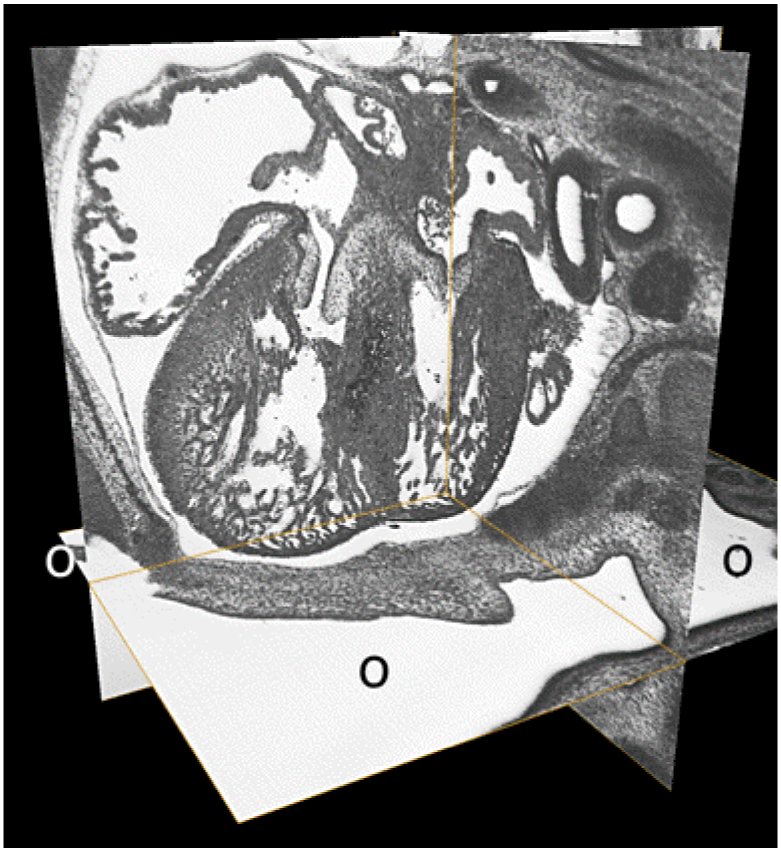

**FIGURE 3.** Three virtual planes, cutting through HREM volume data, showing the heart of an E14 mouse embryo. Shining through artefacts appear to be sufficiently blocked. o, plane of original HREM images.

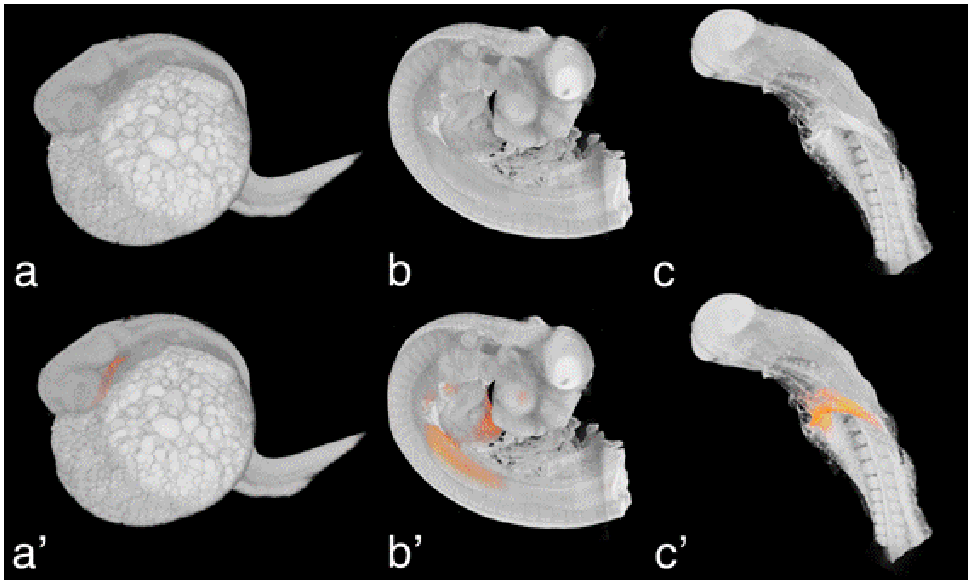

**FIGURE 4.** Volume-rendered 3D computer models. (a,a') Zebrafish embryo (24–28 h). Note the *mlc2* products indicating the heart tube in a'. (b,b') Chick embryo (HH20). Note the *tbx5* products in the left heart. (c,c') Chick embryo (HH 10). Note the *nkx2.5* products.
